# Diagnostic Sensitivity and Specificity of Synovial Biomarkers in Knee Periprosthetic Infections

**DOI:** 10.7759/cureus.91546

**Published:** 2025-09-03

**Authors:** Yusuf Hakan Abaci, Cengiz Yilmaz

**Affiliations:** 1 Orthopaedics and Traumatology, Acibadem Adana Hospital, Adana, TUR; 2 Orthopaedics and Traumatology, Faculty of Medicine, Mersin University, Mersin, TUR

**Keywords:** biomarker, infected knee arthroplasty, infectious disease diagnosis, knee arthroplasty, orthopedic implant-related infection, synovial fluid analysis

## Abstract

Background: Periprosthetic infections are challenging clinical situations in orthopedic surgery. Although it has been investigated by various methods over the years, there is still no clear consensus on diagnosis.

Aim and objective: Our aim in this study is to investigate the diagnostic value of synovial biomarkers, which have gained popularity recently, in periprosthetic infections. Therefore, the diagnostic accuracy of five promising biomarkers in knee periprosthetic infections is evaluated in this study.

Methods: Synovial fluid was obtained by performing sterile puncture from the affected knees of patients with suspected prosthetic knee joint infection and meeting the inclusion criteria, and the material was divided into two aliquots. One of the samples obtained was delivered to the microbiology laboratory; gram staining, direct microscopic examination, and cell count were performed, and a culture study was carried out by incubating for five days. Eosin-methylene blue (EMB) agar, blood agar, and Sabouraud dextrose agar (SDA) were used for culture. According to the Musculoskeletal Infection Society (MSIS) criteria, patients were divided into two groups as infected and non-infected. The other sample was delivered to the medical biochemistry laboratory; levels of five biomarkers including bactericidal/permeability-increasing protein-1 (BPI), human neutrophil defensin 1-3 (HNP1-3, alpha-defensins), leukocyte elastase (LE), lactoferrin, and neutrophil gelatinase-associated lipocalin (NGAL) in synovial fluid were studied. BPI, a protein present in granules, has a high affinity for lipopolysaccharides on the bacterial membrane. HNP1-3 is a peptide found in neutrophil azurophilic granules and has a wide spectrum antimicrobial activity. LE is an enzyme deposited in azurophilic granules and has the capability of lysing the cell wall. Lactoferrin is deposited in specific granules and inhibits bacterial growth by binding iron. NGAL is similarly deposited and blocks bacterial iron metabolism. Biomarker levels were analyzed, and the diagnostic value of biomarkers was determined. Patients were also subjected to technetium-99m methylene diphosphonate (Tc-99m MDP) three-phase bone scintigraphy, and the diagnostic value of scintigraphy was assessed.

Results: It was determined that the BPI pg/ml values of infected patients were higher than the values of non-infected patients and that there were statistically significant differences between them (p=0.002). Correlation analysis of biomarkers was done. In the receiver operating characteristic (ROC) curve analysis of the correlation of HNP1-3 with lactoferrin, 40% sensitivity and 100% specificity were detected (positive predictive value (PPV): 70%; negative predictive value (NPV): 100%). The correlation of HNP1-3 with NGAL was found to have 83.3% sensitivity and 100% specificity in the ROC curve analysis (PPV: 50%; NPV: 100%).

Conclusions: The findings of this study differ from those reported in the literature regarding the sensitivity, specificity, and diagnostic accuracy of biomarkers for the diagnosis of periprosthetic infections. However, it was determined in this study that the alpha-defensin-NGAL biomarker combination stands out with its high sensitivity and specificity rate in detecting periprosthetic infection and has a high diagnostic value.

## Introduction

Periprosthetic infection includes septic inflammatory conditions in the tissues surrounding the prosthesis [1]. While infection rates were high in the past, this rate has decreased significantly over time, thanks to advances in antisepsis and sterilization, and current acceptable periprosthetic infection rates range between 0.5% and 2% [2-4]. Although infection is less common than mechanical loosening, it is the most important and most devastating complication [5]. It may lead to prolonged hospitalization, repeated surgical interventions, temporary-permanent implant loss, and shortening of the affected extremity and deformity [5,6].

For years, periprosthetic infections have been diagnosed through procedures requiring various combinations of serologic, microbiologic, histologic, and radiologic studies. Periprosthetic infection is diagnosed by isolating microorganisms from joint fluid samples, surgical wound discharges, or tissue obtained during debridement procedures. Intraoperative tissue sampling is important in order to provide a suitable sample in determining the causative microorganisms, although its sensitivity is low (45-60%) and its specificity is high (96-99%) [7,8].

Despite these expensive and often invasive tests, there is still no consensus on diagnosis. Although the culture has a high specificity, it also has a high rate of false negativity. This makes it challenging to diagnose prosthetic joint infection. Recent studies investigating biomarkers in synovial fluid have gained increasing attention and show promise in improving the diagnosis of periprosthetic joint infections [9,10].

It has been stated that the study of biomarkers in synovial fluid can be used in the diagnosis of periprosthetic infection [11,12]. Shahi and Parvizi studied 16 synovial biomarkers in 2013; they determined the sensitivity and specificity of each, as well as the cutoff values, and stated that biomarkers have high sensitivity and specificity in the diagnosis of prosthetic joint infection [13]. In the biomarker study by Deirmengian et al. which was conducted based on the prosthetic joint infection diagnosis criteria determined by the Musculoskeletal Infection Society (MSIS), striking results were found on five biomarkers (human alpha-defensin 1-3, neutrophil elastase 2, bactericidal/permeability-increasing protein, neutrophil gelatinase-associated lipocalin, and lactoferrin), and it has been suggested that these five biomarkers were 100% sensitive and 100% specific for diagnosing prosthetic joint infection [9].

The aim of this study is to investigate the diagnostic value of synovial biomarkers, which have garnered increasing attention in recent years, in knee periprosthetic infections and to determine their correlation with the MSIS periprosthetic infection criteria.

## Materials and methods

The study was carried out on 28 patients who applied to the outpatient clinic or consulted from other clinics between July 2017 and January 2019 at Mersin University Hospital, Mersin, Turkey. Patients between 50 and 85 years of age who had previously undergone primary or revision knee arthroplasty surgery were evaluated for this study. Inclusion criteria were as follows: clinical suspicion for periprosthetic infection such as pain, swelling, and erythema in the prosthetic knee with high erythrocyte sedimentation rate (ESR) and C-reactive protein (CRP) values ​​in routine blood tests (CRP >9 mg/L or ESR >20 mm/hr); no antibiotic use within the preceding three weeks; completion of three weeks postoperatively; no history of rheumatic disease; and no other inflammatory or infectious disease to increase ESR and CRP values.

Synovial fluid was obtained by aspirating under sterile conditions from the affected knees of patients who met the inclusion criteria. During aspiration, samples were obtained from two separate sites using medial and lateral parapatellar approaches. The obtained synovial fluid was divided into 15 milliliter (ml) sterile polypropylene Falcon (centrifuge) tubes. Samples were delivered to the microbiology laboratory for gram staining of synovial fluid, direct microscopic examination, cell counting, and culture studies. A portion of each sample was separated, resulting in two samples per patient, each of which was inoculated into three cultures. Culture inoculations were done on eosin-methylene blue (EMB) agar, blood agar, and Sabouraud dextrose agar (SDA). The samples were kept in the incubator for five days, and the status of bacterial growth was checked. For biomarker analysis, the remaining two synovial fluid samples were pooled to obtain a single representative specimen per patient. This combined specimen was subsequently transported to the Medical Biochemistry Laboratory, where it was centrifuged at 4000 revolutions per minute (rpm) for 10 minutes. The supernatant part obtained after centrifugation was stored in a -80°C deep freezer until the day of the biomarker study. Specimens were obtained from patients presenting to the outpatient clinic who met the inclusion criteria over an approximate 18-month period, with a total of 28 patients meeting these criteria. On the day of analysis, following thawing of the samples at room temperature, further processing was conducted; bactericidal/permeability-increasing protein-1 (BPI), human neutrophil defensin 1-3 (HNP1-3, alpha-defensins), leukocyte elastase (LE), lactoferrin, and neutrophil gelatinase-associated lipocalin (NGAL) assays were performed using the DSX™ four-plate automated enzyme-linked immunosorbent assay (ELISA) processing system microELISA device (DYNEX Technologies GmbH, Denkendorf, Germany) in accordance with the protocol suggested by the manufacturer. For each analysis, optical density (OD) values ​​whose concentrations correspond to known standards and the curve and equation of the plotted graphics were used, and the concentration of BPI, HNP1-3, lactoferrin, LE, and NGAL was calculated for each sample. Of the biomarkers in the study, human neutrophil defensin (alpha-defensins, HNP1-3) ELISA Kit/96 tests (Hycult; lot number: HK317, Uden, Netherlands) were used for HNP1-3, human lactoferrin ELISA Kit/96 tests (Hycult; lot number: HK329, Uden, Netherlands) were used for lactoferrin, human BPI ELISA Kit/96 tests (Hycult; lot number: HK314, Uden, Netherlands) were used for BPI, human NGAL ELISA Kit/96 tests (Hycult; lot number: HK330, Uden, Netherlands) were used for NGAL, and human LE ELISA Kit/96 tests (MyBioSource; lot number: MBS2602620, San Diego, CA, USA) were used for LE.

The patients were also subjected to scintigraphic evaluations, and signs of infection or aseptic loosening were sought. Technetium-99m methylene diphosphonate (Tc-99m MDP) three-phase bone scintigraphy was used, and increased activity around the prosthesis in all three phases was interpreted as indicative of infection.

Patients were divided into two groups with clinical and microbiological findings, as infected/non-infected, according to the "MSIS Working Group Standard Definition of Periprosthetic Infection" rules. The levels of the five biomarkers mentioned above were studied in the synovial fluid samples obtained from these two groups. The levels and cutoff values ​​of the biomarkers were determined, and their diagnostic power was evaluated in the diagnosis of prosthetic joint infection [9,13]. In addition, diagnostic accuracy evaluation of scintigraphy results was performed between the groups. Approval from the Mersin University Clinical Research Ethics Committee was obtained for the study (approval number: 78017789/050.01.04/390055).

The presence of one of the major criteria or the presence of four of the minor criteria meets the diagnosis of prosthetic joint infection (Table 1). Besides, MSIS stated that if less than four of the minor criteria are found, there may be periprosthetic infection. Also, in infections with low virulence such as *Cutibacterium acnes *(*C. acnes*), although many of these criteria are not met, there may be the presence of periprosthetic infection [14].

**Table 1:** MSIS definition of periprosthetic infection MSIS: Musculoskeletal Infection Society; ESR: erythrocyte sedimentation rate; CRP: C-reactive protein; WBC: white blood cell

MSIS definition of periprosthetic infection [9]	
Major criteria	A sinus tract communicating with the prosthesis
	A pathogen is isolated by culture from two separate tissues or fluid samples obtained from the affected prosthetic joint
Minor criteria	Elevated ESR and CRP (ESR >30 mm/hr; CRP >10 mg/L)
	Elevated synovial fluid WBC count (>3000 cells/µl)
	Elevated synovial fluid neutrophil percentage (>65%)
	Presence of purulence in the affected joint
	Isolation of a microorganism in one periprosthetic tissue or fluid culture >5 neutrophils per high-power field in five high-power fields observed from the histological analysis of periprosthetic tissue at ×400 magnification

IBM SPSS Statistics for Windows, V. 23.0 (IBM Corp., Armonk, NY, USA), package program was used for the statistical analysis of the data obtained from the study. Categorical measurements were summarized as numbers and percentages and continuous measurements as mean and standard deviation (median and minimum-maximum where necessary). Student's t-test and analysis of variance (ANOVA) were used for parameters showing normal distribution according to the number of variables by checking the distributions in the comparison of continuous measurements between the groups. In the study, sensitivity and specificity values ​​were calculated for BPI pg/ml, HNP1-3 pg/ml, LE ng/ml, lactoferrin ng/ml, and NGAL ng/ml biomarkers based on patient groups and the area under the receiver operating characteristic (ROC) curve examined, so the cutoff value is determined. Spearman's rho correlation was applied to determine the differences between biomarker values. Statistical significance level was considered as 0.05 in all tests.

## Results

The mean age of the patients included in the study was 67.75±8.93 years (53-83), the sedimentation value was in the range of 48.82±23.78 mm/hr (14-117 mm/hr), and the CRP values ​​were in the range of 42.37±44.85 mg/L (9-180 mg/L). About 71.4% (n=20) of the patients in the study were female, and 28.6% (n=8) were male. In terms of infection status of the patients, 50% (n=14) were infected, and 50% (n=14) were non-infected according to the MSIS criteria. Around 57.1% of the patients (n=16) were within the first postoperative year, while 42.9% (n=12) were past the first postoperative year. While no growth was observed in culture in 85.7% (n=24) of the patients included in the study, it was found that 14.3% (n=4) had growth in culture. While scintigraphy was performed in 71.4% of the patients (n=20), scintigraphy could not be performed in 28.6% (n=8) due to various reasons (some patients failed to attend their scheduled appointments or were unreachable for follow-up).

When the patient groups were evaluated, no statistically significant linear relationship was observed for age, sedimentation rate, and CRP values ​​between the infected and non-infected groups (p>0.05). While there were statistically significant differences between the groups in terms of culture growth, all four patients with culture growth were in the infected group; methicillin-resistant *Staphylococcus aureus* (MRSA) and methicillin-susceptible *Staphylococcus aureus* (MSSA) pathogens were observed to grow (p<0.05).

There were no statistically significant differences between the groups in terms of HNP1-3 pg/ml, LE ng/ml, lactoferrin ng/ml, and NGAL ng/ml values (p>0.05). It was determined that the BPI pg/ml values ​​of the infected patient group were higher than the values ​​in the non-infected patient group and that there were statistically significant differences between them (p=0.002) (Table 2).

**Table 2:** Biomarker levels in the infected and non-infected groups BPI: bactericidal/permeability-increasing protein-1; HNP1-3: human neutrophil defensin 1-3; LE: leucocyte elastase; Lacto: lactoferrin; NGAL: neutrophil gelatinase-associated lipocalin, pg: picogram; ml: milliliter; ng: nanogram; Avg: average; sd: standard deviation; min: minimum; max: maximum * denotes statistical significance at p<0.05.

Measurements	Non-infected Avg±sd (min-max)	Infected Avg±sd (min-max)	F	P*
BPI pg/ml	17513.45±8237.17 (1905.67-25000.0)	25000±0.0 (25000-25000)	11.565	0.002*
HNP1-3 pg/ml	2427.69±1206.69 (0-4050.33)	2934.73±868.89 (1066.33-4509.0)	1.628	0.213
LE ng/ml	0.43±0.14 (0.30-0.86	0.35±0.12 (0.30-0.80)	2.410	0.133
Lacto ng/ml	89.45±12.29 (57.85-107.87)	81.15±21.76 (28.41-112.73)	1.545	0.225
NGAL ng/ml	5.61±2.67 (1.77-13.22)	5.86±0.61 (4.54-6.60)	0.114	0.738

BPI pg/ml value was found to have a negative moderate relationship with LE ng/ml (r=-0.543) (p<0.05) in the non-infected group (Table 3).

**Table 3:** Correlation analysis of biomarkers by groups *p<0.05; **p<0.01 BPI: bactericidal/permeability-increasing protein-1; HNP1-3: human neutrophil defensin 1-3; LE: leucocyte elastase; Lacto: lactoferrin; NGAL: neutrophil gelatinase-associated lipocalin; pg: picogram; ml: milliliter; ng: nanogram; ESR: erythrocyte sedimentation rate; CRP: C-reactive protein

Measurements			BPI pg/ml	HNP1-3 pg/ml	LE ng/ml	Lacto ng/ml	NGAL ng/ml	CRP	ESR
Non-infected group	BPI pg/ml	r	1	0.356	-0.543^*^	0.254	0.347	0.019	0.441
		p	-	0.212	0.045	0.381	0.225	0.948	0.114
	HNP1-3 pg/ml	r	0.356	1	-0.130	0.558^*^	0.176	-0.320	-0.368
		p	0.212	-	0.657	0.038	0.546	0.265	0.196
	LE ng/ml	r	-0.543^*^	-0.130	1	0.339	0.506	-0.088	0.140
		p	0.045	0.657	-	0.235	0.065	0.766	0.633
	Lacto ng/ml	r	0.254	0.558^*^	0.339	1	0.511	-0.536*	-0.478
		p	0.381	0.038	0.235	-	0.062	0.048	0.084
	NGAL ng/ml	r	0.347	0.176	0.506	0.511	1	0.055	-0.124
		p	0.225	0.546	0.065	0.062	-	0.852	0.673
	CRP	r	0.019	-0.320	-0.088	-0.536*	0.055	1	0.459
		p	0.948	0.265	0.766	0.048	0.852	-	0.099
	ESR	r	-0.441	-0.368	0.140	-0.478	-0.124	0.459	1
		p	0.114	0.196	0.633	0.084	0.673	0.099	-
Infected group	BPI pg/ml	r	-	-	-	-	-	-	-
		p	-	-	-	-	-	-	-
	HNP1-3 pg/ml	r	-	1	0.487	0.583^*^	0.708^**^	-0.512	-0.447
		p	-	-	0.077	0.029	0.005	0.061	0.109
	LE ng/ml	r	-	0.487	1	0.248	0.162	-0.224	-0.361
		p	-	0.077	-	0.392	0.579	0.441	0.205
	Lacto ng/ml	r	-	0.583^*^	0.248	1	0.384	-0.286	-0.193
		p	-	0.029	0.392	-	0.175	0.322	0.509
	NGAL ng/ml	r	-	0.708^**^	0.162	0.384	1	-0.177	-0.001
		p	-	0.005	0.579	0.175	-	0.545	0.999
	CRP	r	-	0.512	-0.224	-0.286	-0.177	1	0.664**
		p	-	0.061	0.441	0.322	0.545	-	0.010
	ESR	r	-	-0.447	-0.361	-0.193	-0.001	0.664**	1
		p	-	0.109	0.205	0.509	0.999	0.010	-

When the correlation distributions of the patients in the infected group were examined, the HNP1-3 pg/ml value had a moderate positive relationship with lactoferrin ng/ml (r=0.583) and NGAL ng/ml (r=0.708) values (p<0.05). In the ROC curve analysis of the correlation of HNP1-3 with lactoferrin, 40% sensitivity and 100% specificity were detected (positive predictive value (PPV): 70%; negative predictive value (NPV): 100%). In the ROC curve analysis of the correlation of HNP1-3 with NGAL, 83.3% sensitivity and 100% specificity were determined (PPV: 50%; NPV: 100%) (Table 3).

**Figure 1:**
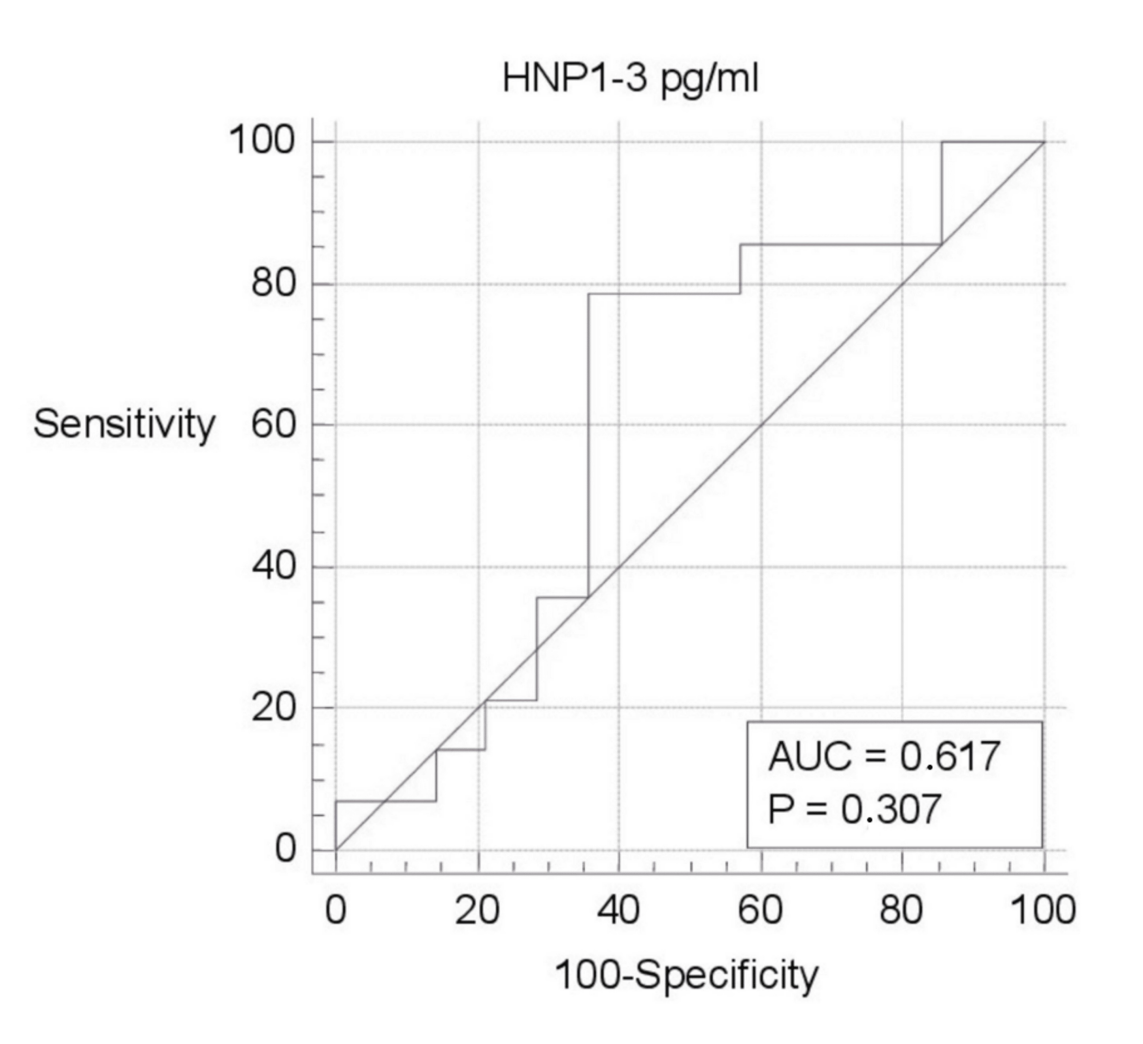
ROC curve analysis of HNP1-3 pg/ml HNP1-3: human neutrophil defensin 1-3; pg/ml: picogram/milliliter; ROC: receiver operating characteristic; AUC: area under the curve

**Figure 2:**
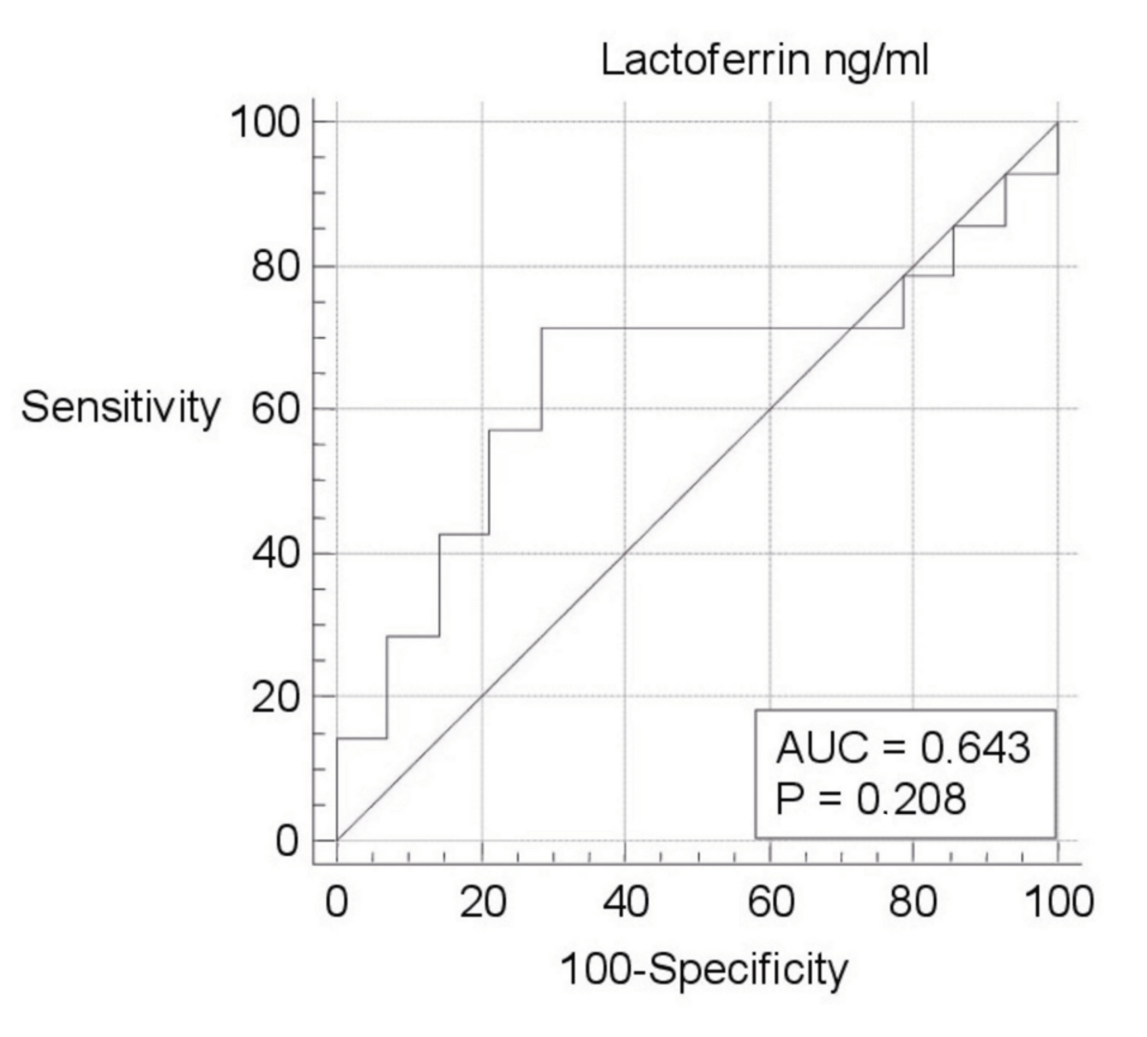
ROC curve analysis of lactoferrin ng/ml ng/ml: nanogram/milliliter; ROC: receiver operating characteristic; AUC: area under the curve

**Figure 3:**
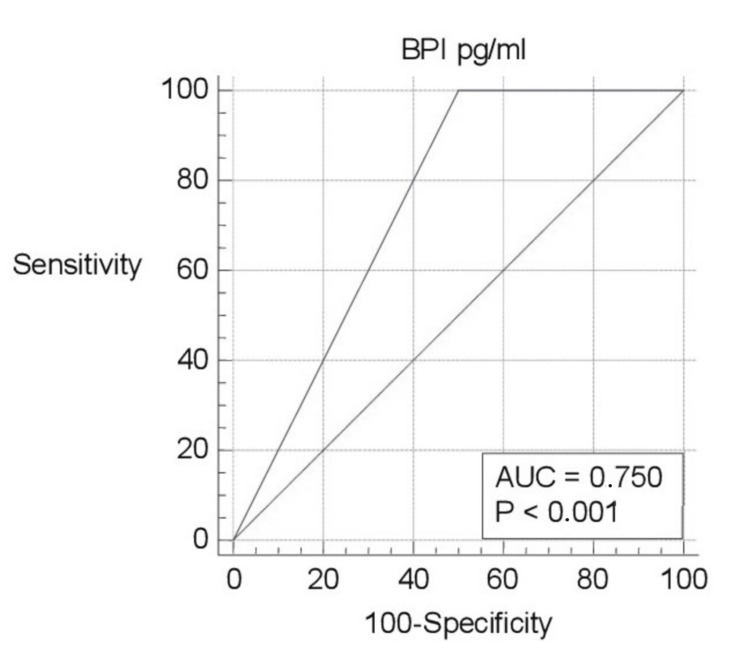
ROC curve analysis of BPI pg/ml pg/ml: picogram/milliliter; BPI: bactericidal/permeability-increasing protein-1; ROC: receiver operating characteristic; AUC: area under the curve

**Figure 4:**
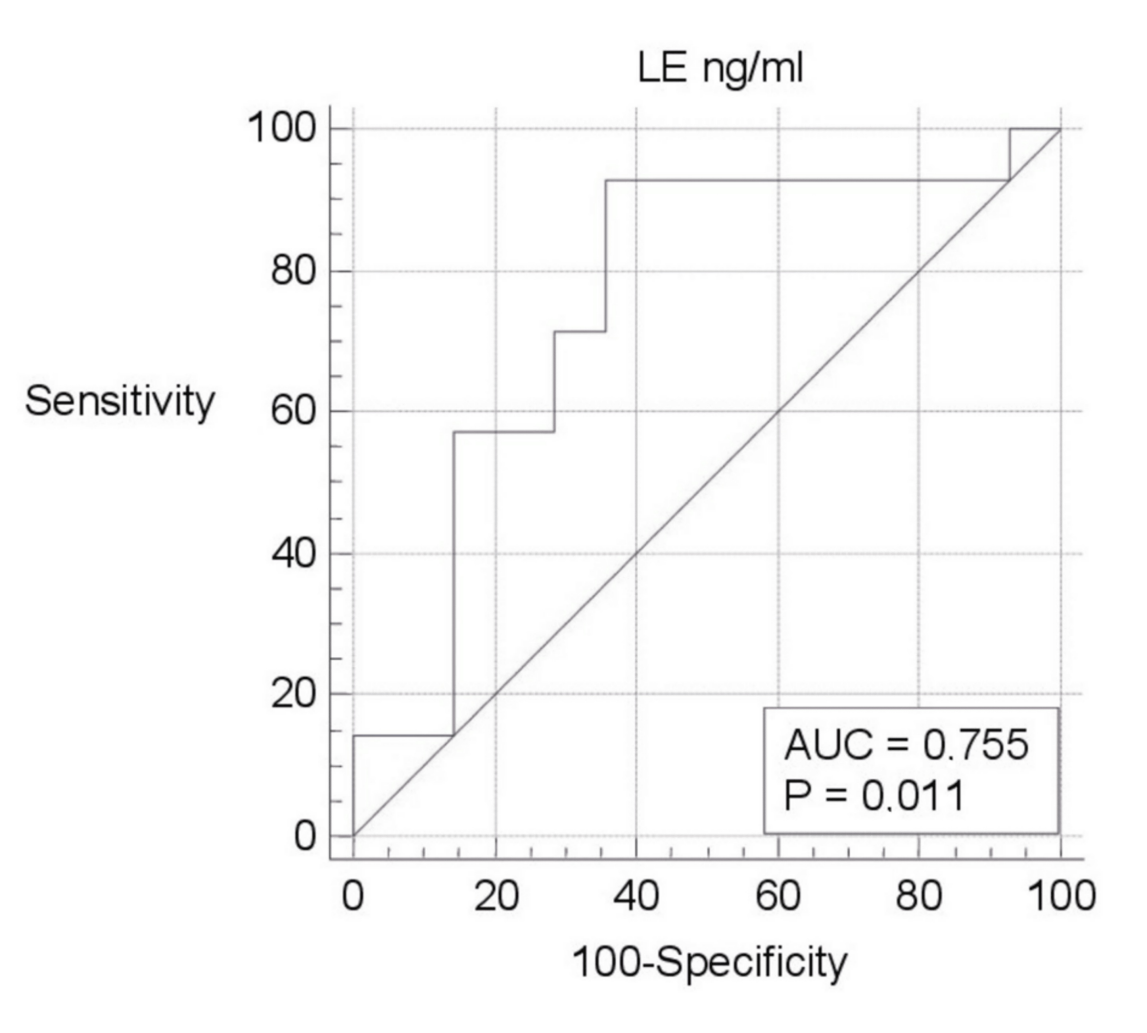
ROC curve analysis of LE ng/ml ng/ml: nanogram/milliliter; LE: leucocyte elastase; ROC: receiver operating characteristic; AUC: area under the curve

**Figure 5:**
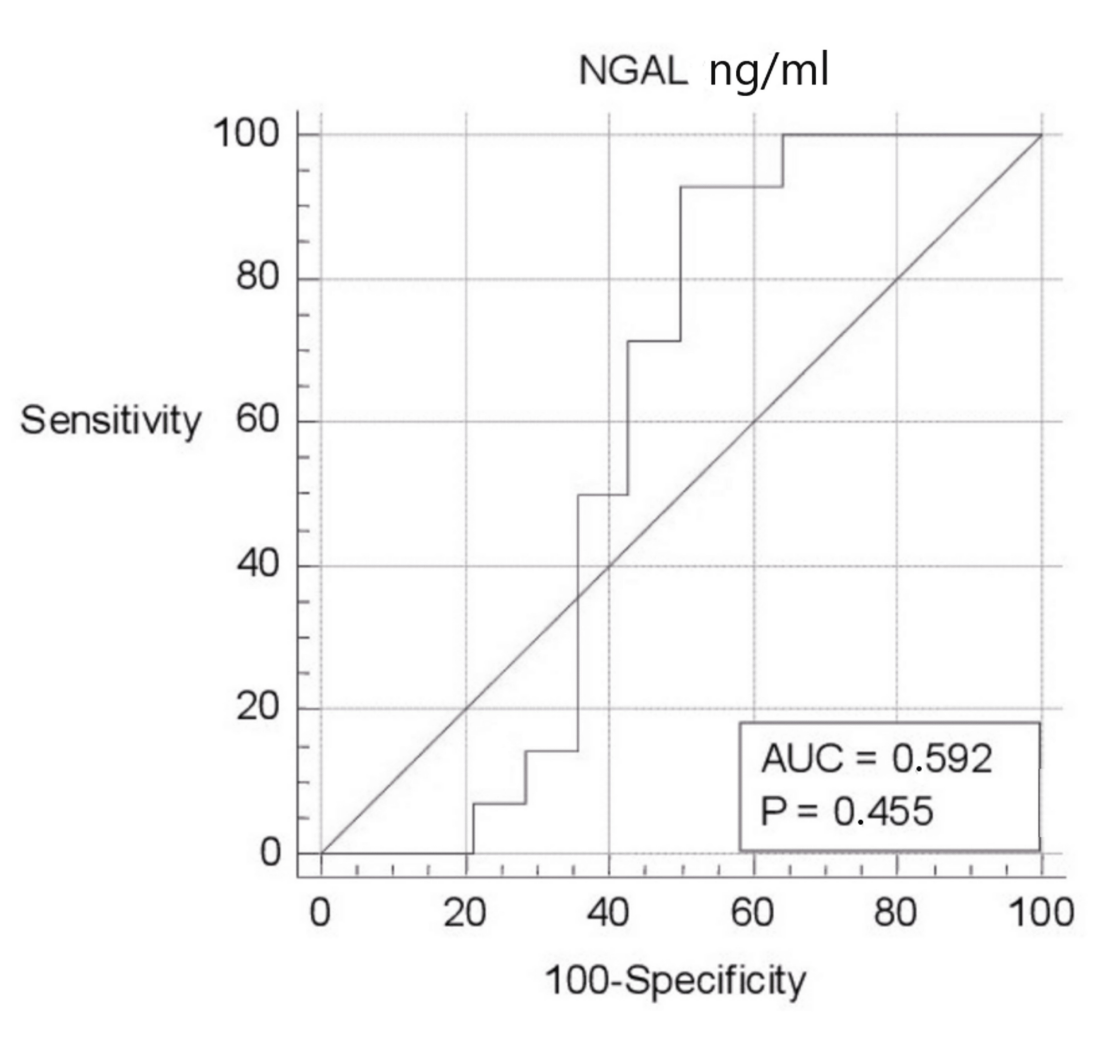
ROC curve analysis of NGAL ng/ml ng/ml: nanogram/milliliter; NGAL: neutrophil gelatinase-associated lipocalin; ROC: receiver operating characteristic; AUC: area under the curve

ROC curve analysis of biomarkers was evaluated separately (Figures 1-5). Area under the curve (AUC) for BPI was 0.750, and the cutoff value was 12682.66 pg/ml; according to this cutoff value, the sensitivity was 100% and the specificity was 50% (p=0.003; PPV: 66.7%; NPV: 100%, with 95%CI). AUC was 0.617, and the cutoff value was 2654 pg/ml for HNP1-3; according to this cutoff value, the sensitivity was 78.57% and the specificity was 64.29% (p=0.307; PPV: 68.7%; NPV: 75%, with 95%CI). AUC was 0.755, and the cutoff value was 0.3544 ng/ml for LE; according to this cutoff value, the sensitivity was 92.86% and the specificity was 64.29% (p=0.011; PPV: 72.2%; NPV: 90%, with 95%CI). AUC was 0.643, and the cutoff value was 88.5 ng/ml for lactoferrin; according to this cutoff value, the sensitivity was 71.43% and the specificity was 71.43% (p=0.208; PPV: 71.4%; NPV: 71.4%, with 95%CI). AUC was 0.592, and the cutoff value was 4.8191 ng/ml for NGAL; according to this cutoff value, the sensitivity was 92.86% and the specificity was 50% (p=0.455; PPV: 65%; NPV: 87.5%, with 95%CI).

## Discussion

In many studies on serum ESR and CRP values, it was stated that sensitivity for the detection of prosthetic joint infection is high, but specificity is low; it has been suggested that they are good markers for exclusion, but they are insufficient for diagnosis [15-18].

Microbiologic culture study is considered the gold standard for periprosthetic infection; however, due to the incubation time, the use of antibiotics, and the formation of biofilms around the prosthesis, it is often not possible to detect bacteria in culture [19]. In the literature, the rate of detecting the pathogen by culture has been found in a wide range of 39-70% [7,19-23]. Although it has been widely reported in the literature that premature antibiotherapy prevents pathogen isolation in culture [24], some studies have suggested that the use of perioperative prophylactic antibiotics has no effect on isolation in culture [25]. Currently, it is recommended to increase the sensitivity and specificity of the culture by extending the incubation time (at least 10 days in case of the presence of slow-growing microorganisms such as *C. acnes*) and by studying samples from at least 3-5 different regions intraoperatively [8,19,20,23,26]. In this study, synovial fluid samples were inoculated onto blood agar, SDA, and EMB agar in the microbiology laboratory, and the average incubation time was five days. In the cultures of 28 patients in this study, growth was observed in four samples (14.3%), MRSA grew in two specimens, and MSSA grew in two specimens. No growth was observed in the culture in any of the samples of the non-infected group, while growth was detected in the culture in four (28.6%) of the 14 samples in the infected group (p<0.05).

Twenty of 28 patients included in this study were subjected to scintigraphic evaluations. Due to the high rate of false positivity in the early postoperative period of scintigraphy [27,28], the patients were divided into two groups as within the first postoperative year and past the first postoperative year. According to the scintigraphic imaging results, there was no statistically significant difference between the patients in the infected and non-infected groups (p>0.05). There was no statistically significant difference in distinguishing infection between the two groups, which were classified as within the first postoperative year and past the first postoperative year.

The primary objective of this study was to evaluate the diagnostic utility of synovial biomarker levels in synovial fluid obtained from patients with suspected prosthetic joint infection. Therefore, five biomarkers that have been previously researched in the literature were selected for the study [9,29-33]. After determining the levels of five biomarkers studied in the synovial fluid obtained from the affected prosthetic joints of the patients, the cutoff value of each was determined by drawing the ROC curve. The diagnostic sensitivities, specificities, PPV, NPV, ​​and safety ranges of biomarkers were determined according to the cutoff value; also, correlation analysis was made between biomarkers.

Alpha-defensin, one of the most prominent biomarkers recently, has been examined; in this study, contrary to the literature, the sensitivity and specificity of alpha-defensin in the diagnosis of periprosthetic infection were found to be quite low. In a retrospective study by Bingham et al. with aspirate samples obtained from 61 joints of 57 patients, it was reported that the sensitivity of alpha-defensin in detecting the infected prosthetic joint was 100% and the specificity was 95% (with 95%CI) [30]. In another study by Deirmengian et al., it was suggested that the diagnostic sensitivity and specificity of not only alpha-defensin but also the other four biomarkers (neutrophil elastase, NGAL, BPI, lactoferrin) mentioned in this study were 100% [9]. In many other studies, the diagnostic sensitivity and specificity of alpha-defensin were found to be markedly higher [13,31,32,34,35]. However, in this study conducted with 28 patients, the diagnostic sensitivity of alpha-defensin was 78.57% and the specificity was 64.29%.

In the biomarker study performed by Li et al. with synovial fluid samples of 50 patients, it was reported that neutrophil elastase stands out with its high sensitivity and specificity in detecting inflammatory joint disease [36]. In addition, the study by Deirmengian et al. reported that elastase has 100% sensitivity and 100% specificity for detecting periprosthetic infection [9]. In this study, 92.86% sensitivity and 64.29% specificity were determined for elastase in detecting periprosthetic infection (p=0.11). Although a statistically significant difference was detected in the ROC curve analysis for elastase, there is a conflict with the literature data in detecting periprosthetic infection, especially in terms of specificity status.

For BPI, 100% sensitivity and 50% specificity were determined; in this study, the levels of BPI between the infected and non-infected groups showed a statistically significant difference in detecting periprosthetic infection (p<0.05). In the study conducted by Deirmengian et al., the diagnostic sensitivity and specificity of BPI were found to be 100% [9].

In the experiment performed by calculating the optimal cutoff value in a series of 72 patients including the control group, the diagnostic sensitivity of lipocalin-2 was found to be 86.3% and its specificity was 77.2%, and lipocalin-2 has been stated to be "nearly perfect" in distinguishing between septic and aseptic loosening [37]. In the study by Deirmengian et al., the sensitivity and specificity of NGAL biomarkers in the diagnosis of prosthetic joint infection were found to be 100% [9]. In this study, 92.86% sensitivity and 50% specificity were determined for NGAL in detecting periprosthetic infection. Although the sensitivity status was compatible with the literature, there were large differences between the specificity status.

Although there is not sufficient data in the literature on lactoferrin, the other biomarker included in this study, there is a great contradiction among the data obtained from the study conducted by Deirmengian et al. which detected 100% sensitivity and specificity for lactoferrin and this study [9]. In this study, the specificity and sensitivity of lactoferrin to diagnose periprosthetic infection were only 71.4%.

It can be hypothesized that the combined use of biomarkers that are evaluated in the infected group and positively correlated in correlation analyses may be useful in diagnosis. When alpha-defensin was evaluated separately with lactoferrin and NGAL, it was observed that the diagnostic power increased and their correlations were statistically significant. The correlation of alpha-defensin with NGAL was found to be statistically significant in diagnosis (p=0.005; p<0.01); in addition, 83.3% sensitivity and 100% specificity were determined in ROC curve analysis of this correlation (PPV: 50%; NPV: 100%). Similarly, the correlation of alpha-defensin and lactoferrin was found to be statistically significant in diagnosis (p=0.029; p<0.05); in the ROC curve analysis of this correlation, 40% sensitivity and 100% specificity were determined (PPV: 70%; NPV: 100%). Although there is no publication in the literature on how the biomarkers used in this study affect the diagnostic power in combination with each other, biomarker combinations with significant diagnostic powers in this study are promising for diagnosis. Especially, the use of alpha-defensin-NGAL biomarker combination, whose correlations have high diagnostic sensitivity and specificity and whose diagnostic power is determined to be highly significant, seems effective in detecting periprosthetic infection.

One limitation of this study is that cutoff values for biomarker levels were determined based on synovial fluid analysis and these values were used to assess the sensitivity and specificity of each biomarker in diagnosing periprosthetic infection. However, the cutoff values identified in this study differed from those reported in the existing literature [9,31,35]. In our study, ESR and CRP were routinely studied in the blood of patients with suspicion of prosthetic joint infection. No statistically significant difference was found in diagnosis for the measured ESR and CRP values ​​between the infected and non-infected patient groups determined according to the MSIS criteria (p>0.05). Besides, only patients with high sedimentation and CRP values ​​were included in this study. Another limitation of this study may be that patients with systemic inflammatory disease, on antibiotic therapy, and who were in the early postoperative period (<3 weeks) were not included. In the study by Deirmengian et al., patients with antibiotic use and systemic disease were also included, but patients in the early postoperative period were not included [9]. However, excluding patients in the early postoperative period is due to the early clinical swelling and erythema and also the high false positivity rate of ESR and CRP values [38]. Another limitation of this study may be the number of patients. Although there is a large patient group in various scientific publications and meta-analyses, this study was conducted with samples from 28 patients [9,30,33,39]. Nevertheless, a power analysis was performed for this study, and the minimum number of patients required was 17. Another limitation of this study may be that patients were grouped according to the MSIS criteria, which is considered the gold standard in periprosthetic infections, thus allowing comparison between the levels of synovial fluid biomarkers of two patient groups (infected and not infected). However, recognizing the infection according to the MSIS criteria, which is widely accepted in prosthetic joint infections, and determining sensitivity and specificity accordingly may also affect the study results and be misleading. Although these criteria are widely accepted, their place in making a definitive diagnosis is still controversial.

This study was conducted using the then-current MSIS criteria, which classified patients as "infected" or "not infected". These criteria were revised in 2018 [40]. However, according to the 2018 MSIS criteria, alpha-defensin was considered a minor criterion for periprosthetic infections. This contradicts the results of our study and needs further investigation.

## Conclusions

Although there have been promising developments in the diagnosis of periprosthetic infection in recent years, a definitive and effective diagnostic method has not been established yet; further prospective randomized controlled studies are needed. However, it was found in this study that the alpha-defensin-NGAL biomarker combination stands out with its high sensitivity and specificity rate in detecting periprosthetic infection and has a high diagnostic value.
